# Supplementary material for: PET Glycolysis to BHET Efficiently Catalyzed by Stable and Recyclable Pd-Cu/γ-Al2O3
Source: Molecules. 2024 Sep 11;29(18):4305. doi: 10.3390/molecules29184305 (PMC11434405; doi:10.3390/molecules29184305)
Supplement: Supplementary file 1 [file molecules-29-04305-s001.zip › molecules-3193176-supplementary.pdf]

PET glycolysis to BHET efficiently catalyzed by stable and recyclable Pd-Cu/γ-Al_2O_3

Supplementary Information

[1] School of Chemical Engineering, Yangzhou Polytechnic Institute, Yangzhou 225127, Jiangsu, China; 17372976062@163.com (E.Q.); Huangh0825@163.com (H.H.); 18012336591@163.com (Y.W.)

[2] Jiangsu Polyester Synthesis and Renewable Technology Engineering Research Center, Yangzhou 225127, Jiangsu, China

* Correspondence: zhoulei940528@163.com (L.Z.); 18362929192@163.com (M.L.)

Main product analysis

The DSC curve of main product was depicted in Figure S1. There was a sharp endothermic peak, and the melting onset temperature was 112.5 °C, consistent with the melting point characteristics of BHET.

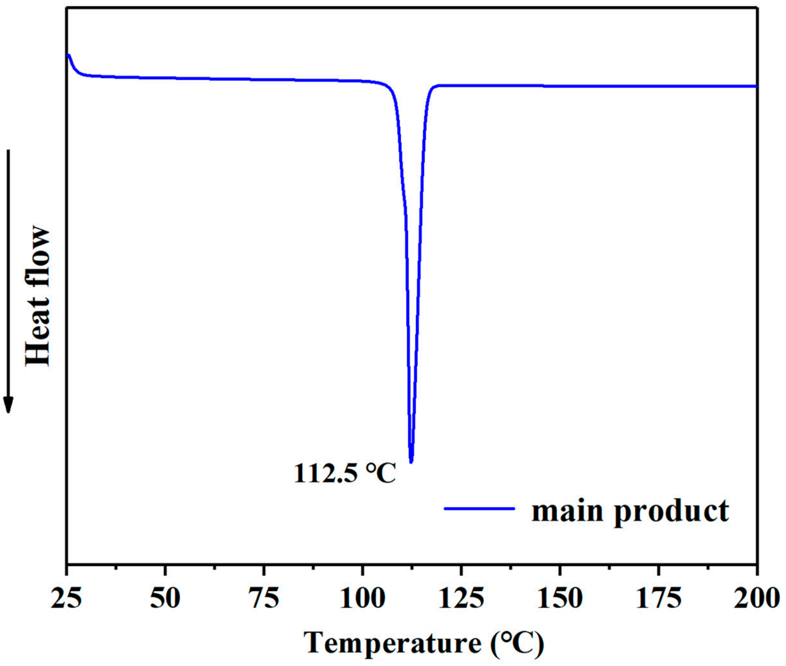

Figure S1. DSC curves of main product

The result of mass spectrometry was shown in Figure S2, MS (m/z): $[M]^+$ calcd for $[BHET+Na]^+$, 277.07; found, 277.10. Further confirmed that the product is BHET.

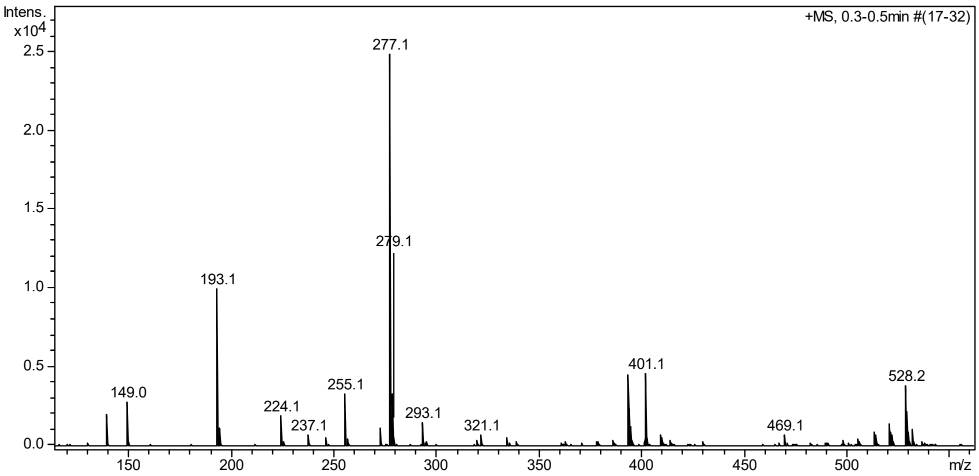

Figure S2. Mass spectrum of main product

The result of ^1H NMR was shown in Figure S3, ^1H NMR (DMSO, 400 MHz), δ 8.12 (s, 4H, Ar), 4.98 (s, 2H, OH), 4.39 – 4.22 (m, 4H, CH_2), 3.73 (q, 4H, CH_2).

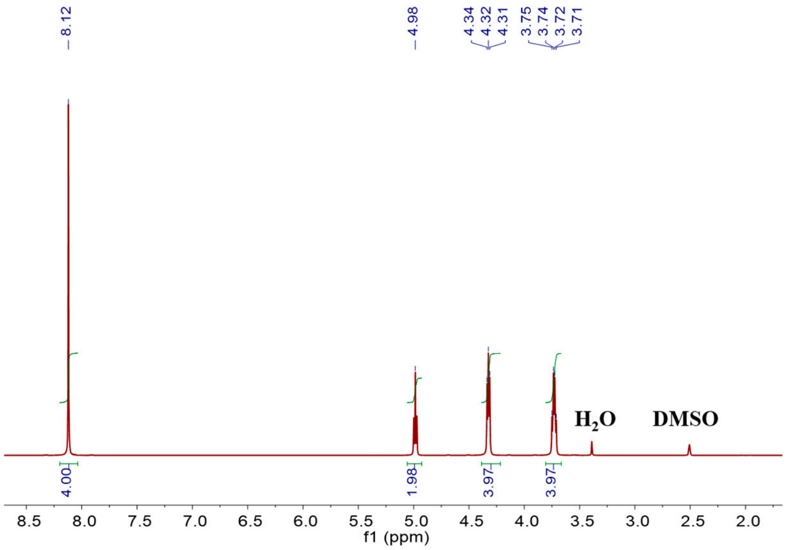

Figure S3. ^1H NMR spectrum of the main product of PET glycolysis in DMSO

Table S1. Elemental analysis results of the main product

	C %	H %	O %
Theoretical value	56.69	5.55	37.76
Main product	56.74	5.62	37.88

Quantitative analysis by HPLC

The yield of the product was determined by external standard method of high-performance liquid chromatography (HPLC). A series of concentration standard BHET solutions were prepared, and the standard curve was obtained according to the peak area of different concentration as shown in Figure S4. The product was confirmed by the same retention time of the HPLC as shown in Figure S5, and the concentration of the sample was calculated according to the value of its peak area into the standard curve, then the yield was obtained.

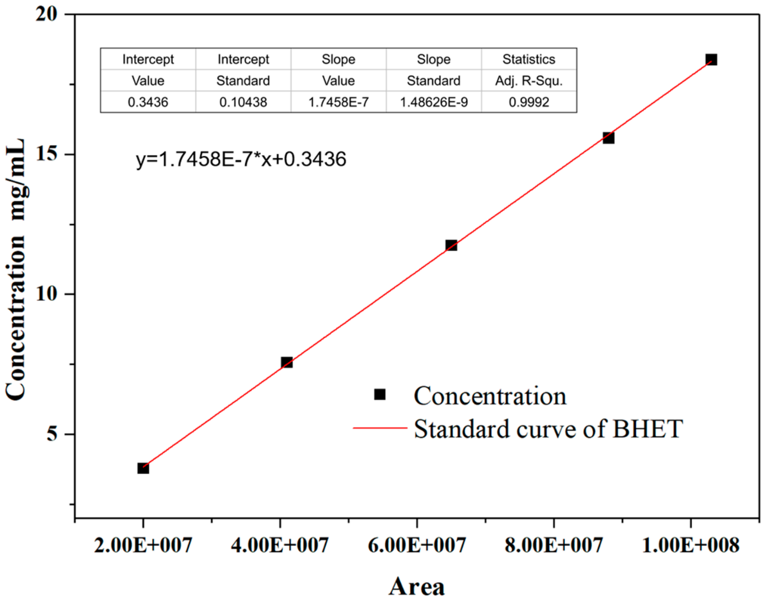

Figure S4. The standard curve of BHET

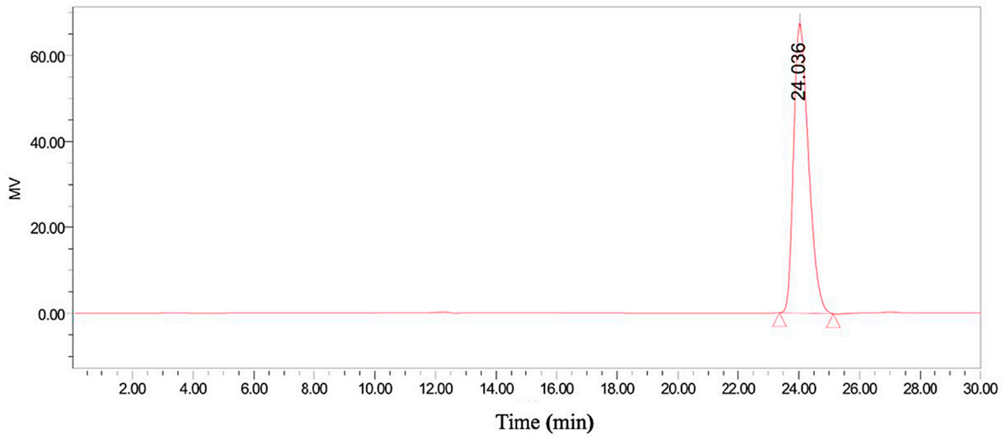

Figure S5. The HPLC chromatogram of main product
